# Bicontinuous Nanophasic Conetworks of Polystyrene with Poly(dimethylsiloxane) and Divinylbenzene: From Macrocrosslinked to Hypercrosslinked Double-Hydrophobic Conetworks and Their Organogels with Solvent-Selective Swelling

**DOI:** 10.3390/gels11050318

**Published:** 2025-04-24

**Authors:** Anna Petróczy, István Szanka, András Wacha, Zoltán Varga, Yi Thomann, Ralf Thomann, Rolf Mülhaupt, Laura Bereczki, Nóra Hegyesi, Béla Iván

**Affiliations:** 1 Polymer Chemistry and Physics Research Group, Institute of Materials and Environmental Chemistry, HUN-REN Research Centre for Natural Sciences, Magyar tudósok körútja 2, H-1117 Budapest, Hungary; szanka.istvan@ttk.hu (I.S.); hegyesi.nora@ttk.hu (N.H.); 2 George Hevesy PhD School of Chemistry, Institute of Chemistry, Faculty of Science, Eötvös Loránd University, Pázmány Péter Sétány 2, H-1117 Budapest, Hungary; 3 Biological Nanochemistry Research Group, Institute of Materials and Environmental Chemistry, HUN-REN Research Centre for Natural Sciences, Magyar tudósok körútja 2, H-1117 Budapest, Hungary; wacha.andras@ttk.hu (A.W.); varga.zoltan@ttk.hu (Z.V.); 4 Freiburg Materials Research Center, University of Freiburg, Stefan-Meier-Str. 21, D-79104 Freiburg, Germany; yi.thomann@fmf.uni-freiburg.de (Y.T.); ralf.thomann@fmf.uni-freiburg.de (R.T.); rolfmuelhaupt@web.de (R.M.); 5 Freiburg Center for Interactive Materials and Bioinspired Technologies (FIT), University of Freiburg, Georges-Köhler-Allee 105, D-79110 Freiburg, Germany; 6 Institute for Macromolecular Chemistry, University of Freiburg, Stefan-Meier-Str. 31, D-79104 Freiburg, Germany; 7 Chemical Crystallography Research Laboratory, Centre of Structural Science, HUN-REN Research Centre for Natural Sciences, Magyar tudósok körútja 2, H-1117 Budapest, Hungary; nagyne.bereczki.laura@ttk.hu; 8 Department of Physical Chemistry and Materials Science, Budapest University of Technology and Economics, Műegyetem rkp. 3, H-1111 Budapest, Hungary

**Keywords:** polystyrene, poly(dimethylsiloxane), conetwork, bicontinuous nanophase separation, double-hydrophobic, hypercrosslinked, organogel, solvent-selective swelling

## Abstract

Polymer conetworks, which consist of two or more covalently crosslinked polymer chains, not only combine the individual characteristics of their components, but possess various unique structural features and properties as well. In this study, we report on the successful synthesis of a library of polystyrene-*l*-poly(dimethylsiloxane) (PSt-*l*-PDMS) (“*l*” stands for “*linked by*”) and polystyrene-*l*-poly(dimethylsiloxane)/divinylbenzene (PSt-*l*-PDMS/DVB) polymer conetworks. These conetworks were prepared via free radical copolymerization of styrene (St) with methacryloxypropyl-telechelic poly(dimethylsiloxane) (MA-PDMS-MA) as macromolecular crosslinker in the absence and presence of DVB with 36:1 and 5:1 St/DVB ratios (*m*/*m*), the latter leading to hypercrosslinked conetworks. Macroscopically homogeneous, transparent conetworks with high gel fractions were obtained over a wide range of PDMS contents from 30 to 80 *m*/*m*%. The composition of the conetworks determined by elemental analysis was found to be in good agreement with that obtained from the ^1^H NMR spectra of the extraction residues, as a new method which can be widely used to easily determine the composition of multicomponent networks and gels. DSC, SAXS, and AFM measurements clearly indicate bicontinuous disordered nanophase separated morphology for all the investigated conetworks with domain sizes in the range of 3–30 nm, even for the hypercrosslinked PSt-*l*-PDMS/DVB conetworks with extremely high crosslinking density. The cocontinuous morphology is also proved by selective, composition-dependent uniform swelling in hexane for the PDMS and in 1-nitropropane for the PSt domains. The Korsmeyer–Peppas type evaluation of the swelling data indicates hindered Fickian diffusion of both solvents in the conetwork organogels. The unique nanophasic bicontinuous morphology and the selective swelling behavior of the PSt-*l*-PDMS and PSt-*l*-PDMS/DVB conetworks and their gels offer a range of various potential applications.

## 1. Introduction

Polymer conetworks, composed of different polymer chains linked by covalent bonds to each other, have gained significant interest due to their unique structural features and properties, as well as to their broad variety of special application possibilities, ranging from biomedicine to nanotechnologies, coatings, sensors, catalyst supports, nanohybrids, ophthalmic devices, sorbents, membranes, etc. (see e.g., Refs. [1,2,3,4,5,6,7,8,9,10,11,12,13,14,15,16,17,18,19,20,21,22,23,24,25,26,27,28,29,30,31,32,33,34,35,36,37,38,39,40,41,42,43,44,45,46,47,48,49,50,51,52,53,54,55,56,57,58,59,60,61,62,63,64,65,66,67] and references therein). Such macromolecular assemblies have mainly been prepared by the so-called macromonomer method or by chain–chain coupling processes. The macromonomer method applies polymers with two or more polymerizable groups, acting as macromolecular crosslinkers (macrocrosslinkers) in copolymerizations with selected monomers [1,2,8,9,10,11,12,13,14,15,16,17,18,19,20,21,32,33,34,35,36,37,51,52,53,54,55,56,57,58,59,60,61,62,63]. Chain–chain coupling can lead to conetworks by reacting polymers with each other having suitable terminal and/or pendant functional groups [1,4,7,22,23,24,25,26,27,28,29,38,39,40,41,42,43,44,45,46,47,48,49,50,64,65,66,67,68,69,70,71,72,73,74]. When hydrophilic and hydrophobic chains are crosslinked, amphiphilic conetworks are obtained [1,2,3,4,5,6,7,8,9,10,11,12,13,14,15,16,17,18,19,20,21,30,31,32,33,34,35,36,37,38,39,40,41,42,43,44,45,46,47,48,49,50,51,52,53,54,55,56,57,58,59,60,61]. Network formation by linking hydrophilic polymers with other hydrophilic chains or by crosslinking hydrophobic macromolecules with other hydrophobic ones results in double-hydrophilic [62,63,64,75,76] or double-hydrophobic [22,23,24,25,26,27,28,29,72] conetworks, respectively. When immiscible polymer chains form the conetworks, usually disordered nanophase-separated morphology exists in these materials with broad bicontinuous (cocontinuous) composition window [1,9,10,14,19,21,59,77,78,79,80,81,82,83]. This nanophasic spatial arrangement of the components in such conetworks results in a variety of unique surface and bulk properties, which enable a broad range of special applications as mentioned above.

Among the hydrophobic components applied so far, poly(dimethylsiloxane) (PDMS) with functional groups is frequently used for the preparation of amphiphilic conetworks by either the macromonomer method [9,10,11,12,13,14,15,16,17,18,19,20,21,32,33,34,35,36,37,51,52,53,54,55,56,57,58] or chain–chain coupling processes [7,22,23,24,25,26,27,28,29,38,39,40,41,42,43,44,45,46,47,48,49,50]. This is mainly due to the advantageous properties of PDMS, such as biocompatibility, high oxygen permeability, optical transmittance, good mechanical properties, and thermal stability. These features offer diverse application possibilities for PDMS-based conetworks, for instance, ophthalmic devices [3,4,5,6,7,8], phase transfer matrices [9,10], separation membranes [11,12,13,14,15], coatings [16,17,34,52], sensors [18,19,20,21,35], precursors for porous materials [22,23,24,25,26,27,28,29], catalysis [30,31,32,33], thermal energy storage materials [43], wearable energy transfer devices [54,55], immunoisolation membranes [56], etc.

It is interesting to note that while functional PDMS have been widely used in silicone rubbers and various crosslinked PDMS (see, e.g., Refs. [84,85,86,87] and references therein), and PDMS chains are incorporated into various amphiphilic conetworks, only few reports exist on PDMS-containing hydrophobic–hydrophobic conetworks [22,23,24,25,26,27,28,29,88,89,90,91,92,93]. Among the various choices of hydrophobic polymers, the combination of polystyrene (PSt) with PDMS in conetworks is expected to lead to materials with remarkable structural and property features, due to the known immiscibility of PSt and PDMS [89,93,94,95]. Surprisingly, however, studies on conetworks with PSt as one of the components have been quite rarely reported so far [88,89,90,91,92,93,96,97,98,99,100], despite the fact that polystyrene crosslinked or even hypercrosslinked with divinylbenzene (DVB) has been used for various applications for a long time (see e.g., Refs. [100,101,102,103,104,105,106] and references therein), on the one hand. On the other hand, only few reports exist on conetworks consisting of polystyrene and poly(dimethylsiloxane) as major covalently coupled components without detailed composition, structure and swelling characterization [88,89,90,91,92,93].

In this study, we present the synthesis of a library of polystyrene-*l*-poly(dimethylsiloxane) (PSt-*l*-PDMS) and polystyrene-*l*-poly(dimethylsiloxane)/divinyl-benzene (PSt-*l*-PDMS/DVB) double-hydrophobic conetworks with a broad range of composition and with different St/DVB ratios on conetwork formation, structure and swelling behavior. Methacryloxypropyl–telechelic PDMS (MA-PDMS-MA) is used as a macrocrosslinker in radical copolymerization with styrene for the synthesis of conetworks in the absence and presence of DVB as an additional low-molecular-weight crosslinker with 36:1 and 5:1 (*m*/*m*) St/DVB ratios, in order to systematically investigate the effect of DVB on the formation and properties of conetworks with PSt and PDMS macromolecular components. The applied St/DVB ratios of 1:0, 36:1 and 5:1 (*m*/*m*) are expected to yield exclusively macrocrosslinked, moderately crosslinked, and hypercrosslinked conetworks with unprecedented broad range of compositions, respectively. The resulting conetworks were investigated for gel fractions of the syntheses, composition of the conetworks after extraction of the unreacted species, morphology determination by DSC, SAXS, and AFM, and as well as for their swelling behavior in selective solvents for the components.

## 2. Results and Discussion

### 2.1. Synthesis of PSt-l-PDMS and PSt-l-PDMS/DVB Polymer Conetworks

**Scheme 1:**
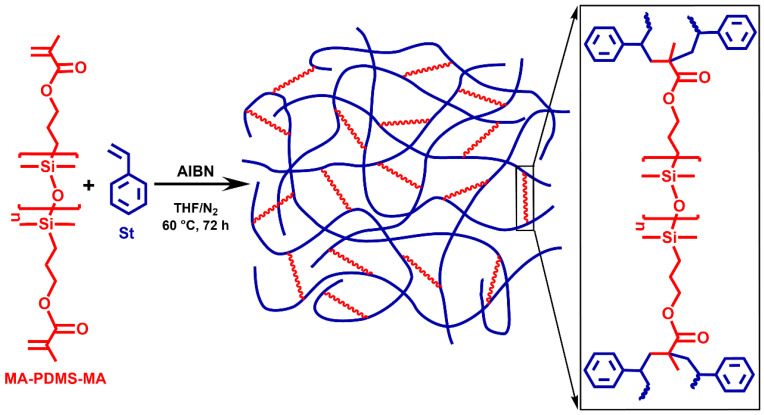
The synthesis of polystyrene-*l*-poly(dimethylsiloxane) (PSt-*l*-PDMS) polymer conetworks and the structure of the crosslinking sites by the MA-PDMS-MA macrocrosslinker.

**Scheme 2:**
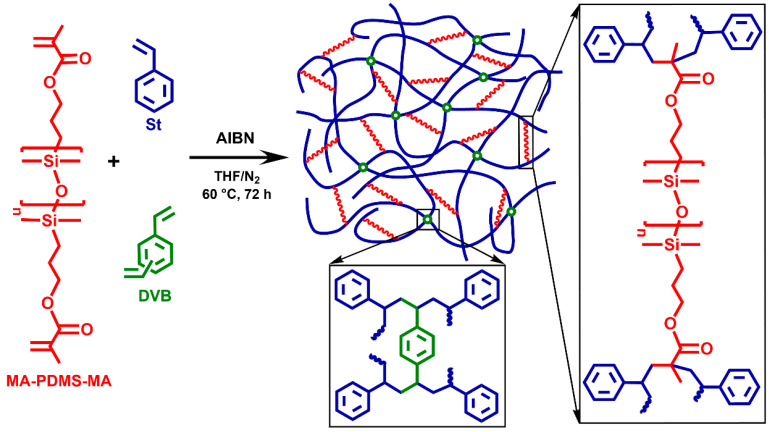
The synthesis of polystyrene-*l*-poly(dimethylsiloxane)/divinylbenzene (PSt-*l*-PDMS/DVB) polymer conetworks and the structure of the crosslinking points by DVB and the methacryloxypropyl–telechelic PDMS (MA-PDMS-MA) macrocrosslinker.

The polystyrene-*l*-poly(dimethylsiloxane) (PSt-*l*-PDMS) conetworks were prepared by the so-called macromonomer method via free radical copolymerization of styrene (St) with methacryloxypropyl–telechelic poly(dimethylsiloxane) (MA-PDMS-MA) (*M*_n_ = 4700 g/mol) serving as macromolecular crosslinker as displayed in Scheme 1 (“*l*” stands for “*linked by*”). In order to investigate the effect of divinylbenzene (DVB), which is a conventional crosslinker in polystyrene networks, polystyrene-*l*-poly(dimethylsiloxane)/divinylbenzene (PSt-*l*-PDMS/DVB) conetworks with additional DVB crosslinkers were also synthesized (Scheme 2). Two series of conetworks were prepared in the presence of DVB with 36:1 and 5:1 St/DVB ratios (*m*/*m*). In the case of the higher DVB content, i.e., with the 5:1 St/DVB ratio, hypercrosslinked conetworks were expected. For both the PSt-*l*-PDMS and PSt-*l*-PDMS/DVB series, the PDMS content in the feed was varied in the range of 30–70 wt%, resulting in a library of conetworks with a broad composition range. All polymerizations were carried out in THF, a common solvent for the starting materials, the initiator and the polystyrene homopolymer, ensuring a homogeneous starting solution and fully swollen gels during the copolymerization reaction (see Tables S1–S3 for feed compositions in the Supplementary Materials). Taking into account that the copolymerization reactivity ratios of styrene and methyl methacrylate are close to each other in the range of 0.5 (see e.g., Refs. [107,108] and references therein), random crosslinking between styrene and the methacrylate–telechelic MA-PDMS-MA is expected in the course of the conetwork formation under the applied conditions.

**Figure 1:**
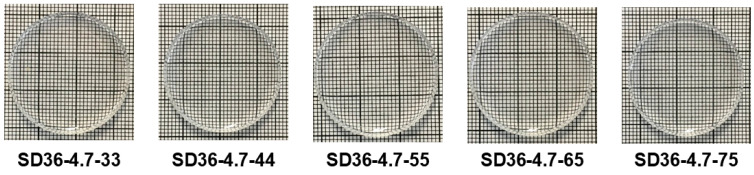
The transparent PSt-*l*-PDMS/DVB conetworks with 36:1 St/DVB weight ratio.

The conetwork forming reaction by the copolymerization of styrene with the MA-PDMS-MA macrocrosslinker successfully resulted in conetworks with reasonably high gel fractions in the range of 53–63% as presented in Table 1. The use of the highly reactive DVB as additional crosslinker leads to higher gel fractions in the range of 84–92% (Table 1). As displayed in Figure 1, all the conetworks are fully transparent materials regardless of their composition, demonstrating their uniformity and optical clarity.

The composition of the synthesized conetworks was determined by elemental analysis and by evaluating the PDMS content of the extraction residues (determined by ^1^H NMR spectroscopy) and that of the feed ratio. As presented in Table 1, the composition values of the conetworks, determined by these two methods show good agreement. This finding indicates that the composition of two- or multi-component polymer networks, gels or blends can be reliably determined by measuring the composition of the extractables by spectroscopic or chromatographic methods. Applying such a process might be especially important for multicomponent crosslinked polymers where elemental analysis is not sufficient for composition determination, or even in cases when the elemental analysis is unavailable due to its complexity or cost, particularly because the properties of multicomponent polymer networks strongly depend on the composition of such materials.

The data in Table 1 also indicate that the PSt-*l*-PDMS conetworks contain a higher proportion of PDMS than that initially present in the feed. However, when DVB was added, conetworks with compositions close to the feed ratios were obtained. These results indicate that the crosslinker concentration provided solely by methacryloxypropyl–telechelic PDMS (MA-PDMS-MA) is insufficient to achieve higher gel fractions, and soluble fractions with higher polystyrene contents are formed. Addition of DVB increases the crosslinker concentration, and this leads to higher gel fractions. It is noteworthy that the MA-PDMS-MA macrocrosslinker incorporates into the conetworks at high levels, close to the feed ratios, even when a high DVB/St ratio of 1:5 (*m*/*m*) was used. This means that hypercrosslinked conetworks with predetermined macrocrosslinker contents can be obtained in the presence of additional low molecular weight crosslinkers.

### 2.2. Structural Characterization of the PSt-l-PDMS and PSt-l-PDMS/DVB Conetworks

**Figure 2:**
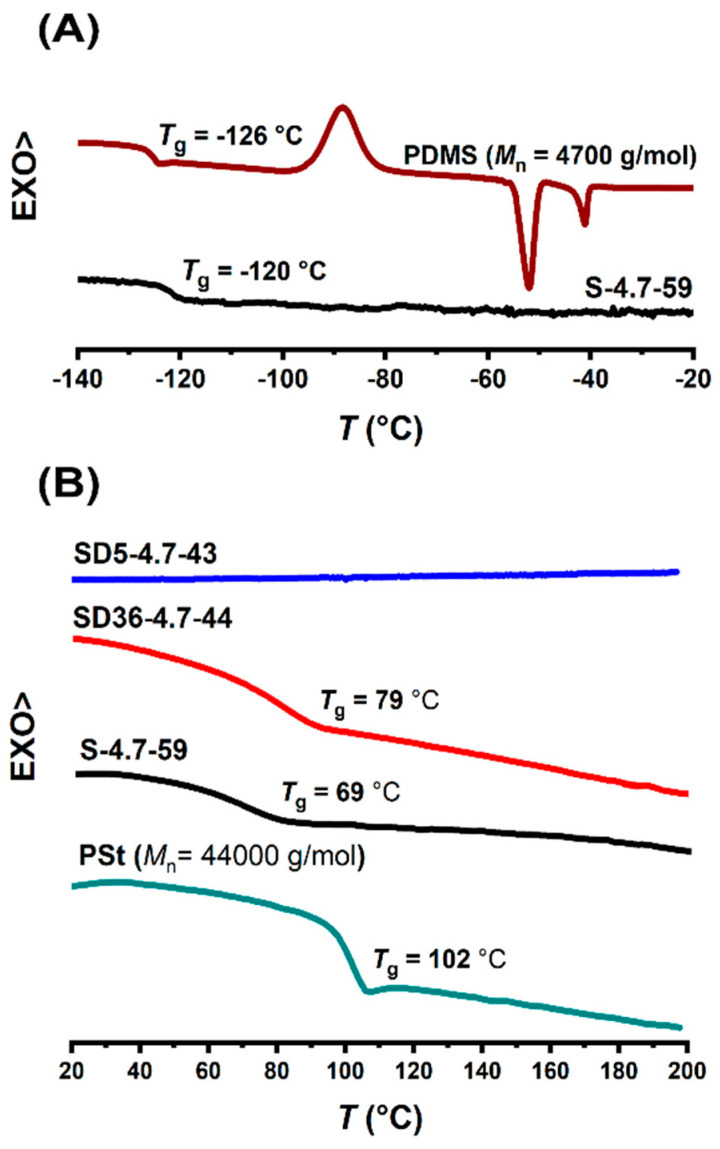
The DSC traces in the ranges of the glass transitions of PDMS (**A**) and PSt (**B**) for the PDMS and PSt homopolymers, and the PSt-*l*-PDMS and PSt-*l*-PDMS/DVB conetworks.

In order to reveal the structural arrangements of the transparent PSt-*l*-PDMS and PSt-*l*-PDMS/DVB conetworks, composed of macroscopically immiscible PSt and PDMS, DSC, SAXS and AFM measurements were carried out. As displayed in Figure 2, the DSC traces indicate glass transition for both the PDMS and PSt components, with the exception of the conetwork with 5:1 St/DVB weight ratio. The absence of glass transition in this case is due to the significantly high crosslinking density. A similar observation was made with all the investigated conetworks. As shown in Figure 2A, the MA-PDMS-MA homopolymer has a glass transition at −126 °C, a cold-crystallization exotherm peak at −70 °C, and a melting endotherm peak at −43 °C, which are consistent with reported data [109]. In the conetwork samples, the glass transition temperature (*T*_g_) of the PDMS component is in the same range (−120 °C). However, the exotherm and endotherm peaks are absent. This indicates that the PDMS chains cannot crystallize within the conetworks. Similar findings, i.e., suppression of the crystallization of semicrystalline components in conetworks, were reported for several other conetworks as well [82,110,111,112], indicating that this is a common phenomenon in polymer conetworks. The DSC curves in the range of the glass transition temperature of the PSt homopolymer are shown in Figure 2B. The *T*_g_s of the polystyrene component in the conetworks were detectable for samples synthesized without DVB or with 36:1 St/DVB weight ratio. The glass transition temperatures of PSt in the conetworks are lower than that of the PSt homopolymer, which is in accordance with results reported for other conetworks prepared by the macromonomer method [83,91,111,112]. As displayed in Figure 2A,B, the DSC curves of the PSt-*l*-PDMS and PSt-*l*-PDMS/DVB conetwork samples (with a 36:1 St/DVB weight ratio) exhibit two distinct glass transitions: one near the *T*_g_ of PDMS and another near to the *T*_g_ of PSt. These results clearly indicate that PDMS and PSt form separate domains within the conetworks. Considering that all conetworks are fully transparent, the sizes of the separate domains are expected to be in the range below the wavelength of the visible light.

**Figure 3:**
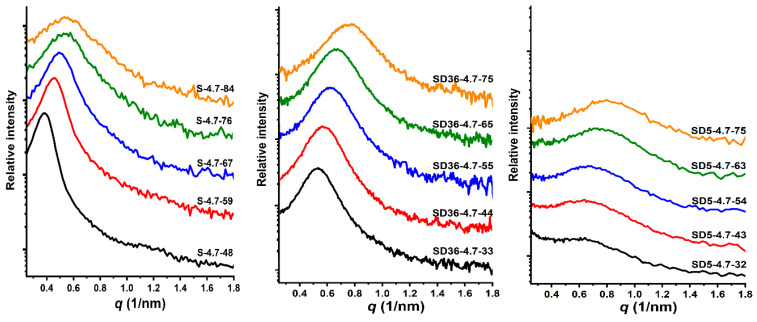
The SAXS curves of the PSt-*l*-PDMS and PSt-*l*-PDMS/DVB conetwork series.

**Figure 4:**
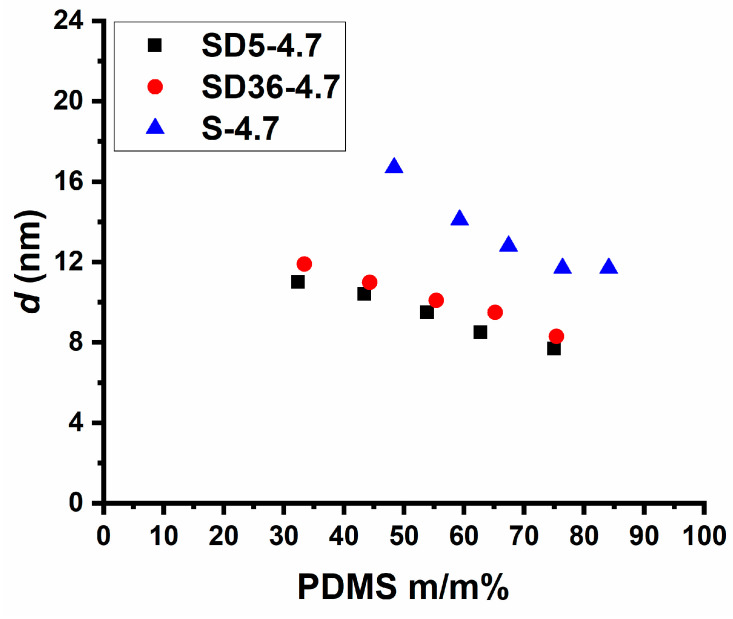
The *d*-spacing values of the PSt-*l*-PDMS and PSt-*l*-PDMS/DVB conetworks obtained by SAXS measurements as a function of the PDMS content.

As shown in Figure 3, the SAXS curves consistently exhibit a single peak, indicative of disordered phase separation within the samples. Notably, the maxima of these curves are shifted towards larger values of the scattering vector (*q*) with increasing PDMS content. Deeper data analysis revealed that the *d*-spacing (average domain spacing), defined as *d* = 2π/*q*_max_, is composition-dependent. For the PSt-*l*-PDMS conetworks, it ranges from 11.7 to 16.7 nm. In contrast, the *d*-spacing values change from 8.3 to 11.9 nm and from 7.9 to 11.2 nm for conetworks with 36:1 and 5:1 St/DVB weight ratios, respectively, as presented in Table 2 and Figure 4. As shown in Figure 3, the shape of the scattering curves becomes broader with increasing the DVB content. This indicates broader distribution of domain distances in the DVB containing conetworks, on the one hand. On the other hand, the *d*-spacing is almost the same for both conetworks containing DVB, while it is higher for the PSt-*l*-PDMS conetworks by ~3–6 nm than that for the PSt-*l*-PDMS/DVB samples. These findings clearly indicate that both the PSt-*l*-PDMS and PSt-*l*-PDMS/DVB conetworks possess nanophase separated morphology with *d*-spacing in the 8–17 nm range, which is consistent with the transparency of all these conetworks, i.e., these conetworks are composed of separate PSt and PDMS nanodomains. The absence of higher order peaks in the SAXS curves indicates the absence of detectable long-range ordering, that is, it can be concluded that all the investigated conetworks possess disordered nanophasic morphology.

**Figure 5:**
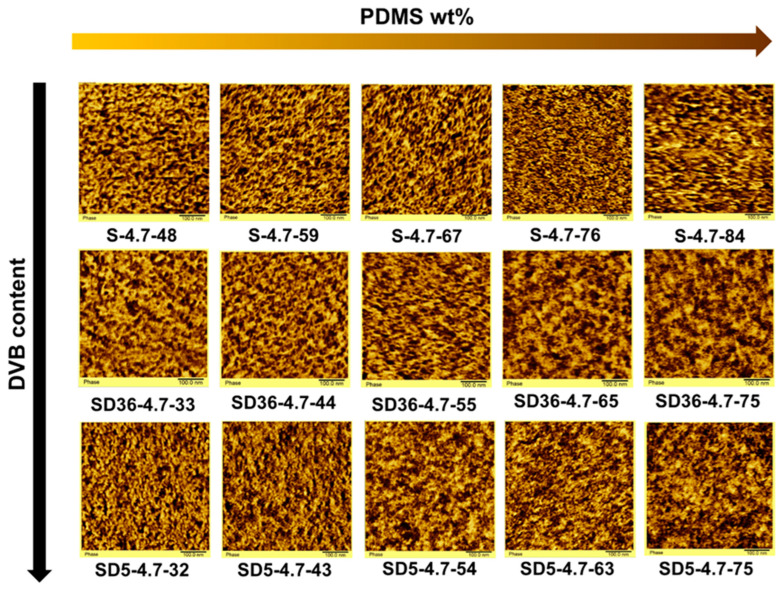
The AFM images of the PSt-*l*-PDMS and PSt-*l*-PDMS/DVB conetworks (the dark areas belong to the PDMS phases, the lighter, yellow color corresponds to the PSt domains; image sizes: 500 × 500 nm).

To visualize the nanophase-separated domain structure of the investigated conetworks, phase imaging AFM measurements were carried out. As displayed in Figure 5, the AFM images show distinct nanophase separation between the PSt and PDMS phases in all conetworks. This is independent of the presence or absence of DVB as an additional low molecular weight crosslinker. This observation corroborates the DSC results on the existence of phase separation, and as well as the SAXS measurements, confirming that the PSt-*l*-PDMS and PSt-*l*-PDMS/DVB conetworks have a nanophase-separated morphology with domain sizes in the ~3–30 nm range in all studied cases. The good contrast of the AFM images enables us to estimate the average domain distances. As presented in Table 2, these values, obtained from the evaluation of the AFM images, are in the range of 9–16 nm, in good agreement with the *d*-spacing results obtained from the SAXS measurements. A closer look at the AFM images reveals four more important features in relation to the nanophasic structure of these conetworks: (1) The AFM images in Figure 5 convincingly show a disordered nanophase separation in all these conetworks, in good agreement with the SAXS curves in Figure 3. These curves have a single scattering maximum, indicating the absence of a higher order (long range order) of the separated nanophases. (2) Bicontinuous (cocontinuous) nanophase-separated morphology exists in all the PSt-*l*-PDMS and PSt-*l*-PDMS/DVB conetworks, independent of the composition. (3) The average domain distances and sizes decrease with the increasing PDMS content of the conetworks, with the exception of the PSt-*l*-PDMS conetworks with the highest PDMS contents (samples S-4.7-84 and SD5-4.7-75), as shown in Figure 4. Similar findings, i.e., a decrease in d-spacing with increasing PDMS content, were recently reported for poly(2-hydroxyethyl acrylate)-*l*-PDMS conetworks [50]. (4) The presence of DVB as an additional low molecular weight crosslinker broadens the domain size distribution, and thus also the domain distance distribution, in the conetworks, confirming the broadening of the SAXS curves for the DVB-containing conetworks as shown in Figure 3. These findings clearly indicate that both the PSt-*l*-PDMS and PSt-*l*-PDMS/DVB conetworks have unique disordered bicontinuous (cocontinuous) nanophase-separated morphology with domain sizes in the range of ~3–30 nm in a broad composition window of 30–80 wt% PDMS content.

### 2.3. Solvent-Selective Swelling of the PSt-l-PDMS and PSt-l-PDMS/DVB Conetwork Gels

**Figure 6:**
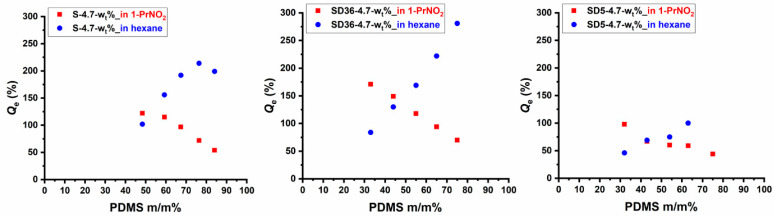
The equilibrium swelling degrees of the PSt-*l*-PDMS and PSt-*l*-PDMS/DVB conetworks in hexane and 1-nitropropane.

The swelling behavior of crosslinked polymers is a critical property which provides insights into their structural characteristics as well. In order to reveal whether a solvent which is only solvent for one of the components in the PSt-*l*-PDMS and PSt-*l*-PDMS/DVB conetworks is able to swell preferentially the given component, detailed swelling experiments were carried out with 1-nitropropane (1-PrNO_2_) and n-hexane. Among these, 1-PrNO_2_ is a good solvent for PSt but is a non-solvent for PDMS, as found during preliminary solubility tests, while hexane is a good solvent for PDMS, but PSt is insoluble in this solvent. As shown in Figures S1 and S2, the swelling degrees were measured as a function of time until equilibrium swelling was achieved for all the samples (with the exception of sample SD5-4.7-75, which disintegrated after a relatively short time when swelling in hexane). As presented in these Figures, both solvents are able to swell the conetworks, and the initial rates of swelling and the equilibrium swelling degrees (*Q*_e_) depend on the composition. The *Q*_e_ values obtained in both 1-PrNO_2_ and n-hexane are displayed in Figure 6 and Figure S3 as a function of PDMS content. The data in these Figures clearly indicate that swelling capacity, i.e., *Q*_e_ decreases in 1-PrNO_2_, while it increases in n-hexane with increasing PDMS content. It can also be seen in Figure S3 that the presence of DVB decreases the equilibrium swelling degrees for a given composition in 1-PrNO_2_ only in the case of conetworks with 5:1 St/DVB (*m*/*m*) ratio, indicating that the PDMS content is the determining factor of *Q*_e_ even at relatively high, that is, at 36:1 St/DVB (*m*/*m*) ratio. In contrast, the *Q*_e_ in hexane decreases with increasing DVB content in the conetworks (Figure S3). The relatively high *Q*_e_ values even at relatively low and high PDMS contents in both solvents confirm the bicontinuous phase separation in these conetworks indicating that the non-swelling phase does not restrict the solvent diffusion in the continuous other phase. As also displayed in Figure 6 and Figure S3, the selective swelling behavior in terms of *Q*_e_ of the PSt-*l*-PDMS and PSt-*l*-PDMS/DVB double-hydrophobic conetwork gels enable preliminary design of such materials with predetermined swelling capacity in the investigated solvents in a wide range of ~20–300% equilibrium swelling degree.

**Figure 7:**
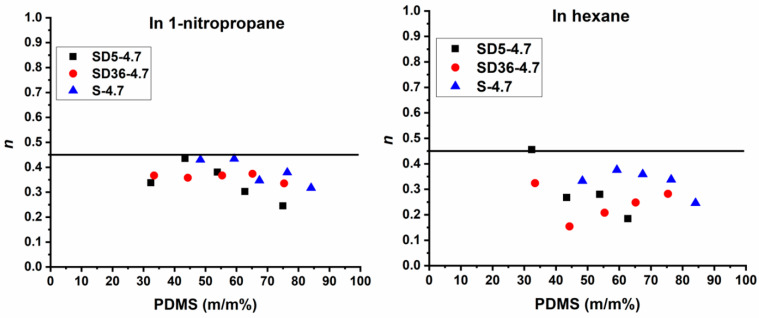
The diffusion exponent (*n*) values of the PSt-*l*-PDMS and PSt-*l*-PDMS/DVB conetworks in 1-nitropropane (1-PrNO_2_) (**left**) and hexane (**right**) as a function of the PDMS content.

Additionally, revealing the swelling mechanism was also attempted by using the Korsmeyer–Peppas relationship [113] between the swelling degree (*Q_t_*) and swelling time (*t*):

(1) $$Q_{t}=k\cdot t^{n}$$

where *k* is a constant and *n* is called as diffusion exponent [113,114,115,116]. The double logarithmic plot of *Q_t_* and the swelling time (*t*) in the initial stage of swelling according to Equation (2), that is,

(2) $${\log(Q}_{t})=\log(k)+n\cdot \log(t)$$

affords the determination of the diffusion exponent. As shown in Figure S4, the log(*Q_t_*) versus log(*t*) plots provide a linear relationship for the swelling in both 1-PrNO_2_ and hexane for all the conetwork samples. The slopes of the straight lines give the diffusion exponent (*n*) values, which are displayed as a function of PDMS contents of the conetworks in Figure 7. In this Figure a line at *n* = 0.45 value indicates Fickian diffusion of the solvent molecules. However, the diffusion exponents range from 0.25 to 0.44 in 1-PrNO_2_ and from 0.15 to 0.46 in hexane, with one exception in the latter case. These results indicate that the diffusion of both solvents during the swelling of either the PSt-*l*-PDMS or PSt-*l*-PDMS/DVB conetworks follows a hindered Fickian diffusion mechanism [116]. This suggests that while solvent diffusion is governed by Fick’s laws, it is restricted by the structural characteristics of the conetworks.

## 3. Conclusions

As presented in this study, a library of novel, fully transparent PSt-*l*-PDMS and PSt-*l*-PDMS/DVB double-hydrophobic conetworks with a broad range of PDMS content (30–80 wt%) have been successfully synthesized by radical copolymerization of styrene with methacryloxypropyl–telechelic PDMS via the so-called macromonomer method in both the absence and presence of DVB as an additional low molecular weight crosslinker. The addition of DVB to the styrene–PDMS reaction mixtures led to higher gel fractions (84–92%) than that for the PSt-*l*-PDMS conetworks (53–63%) due to the increased crosslinking densities caused by the DVB. The lower gel fractions in the case of the PSt-*l*-PDMS conetworks, that is, without the addition of DVB, are presumably due to the formation of a relatively large amount of insufficiently short polystyrene chains for reaching gelation. This is well reflected by the higher PDMS contents in the PSt-*l*-PDMS conetworks than that in the feed, on the one hand. On the other hand, the presence of DVB resulted in conetworks with almost the same composition as that of the styrene and PDMS in the feeds. Relatively high DVB content, i.e., 1:5 (*m*/*m*) DVB/St ratio, led to hypercrosslinked conetworks without preventing the incorporation of the PDMS macrocrosslinker into such conetwork structures.

For the determination of the composition of the conetworks, a new method was developed, which is based on measuring the composition of the extracted (non-gelled) materials by ^1^H NMR spectroscopy. It was found that the composition obtained by elemental analysis of the extracted conetworks and that determined by considering the composition of the extracted materials agree well. This means that when the expensive and laborious elemental analysis of polymer networks, gels, blends, and composites is not available, a well-designed extraction followed by the determination of the composition of the extracted material enables us to obtain the composition of the main polymer product, i.e., the bi- or multi-component polymer system remaining after extraction.

The structural characterization of the transparent, macroscopically homogeneous PSt-*l*-PDMS and PSt-*l*-PDMS/DVB conetworks by DSC, SAXS, and AFM has revealed that a disordered bicontinuous (cocontinuous) nanophase-separated morphology exists in these macromolecular assemblies, which are composed of immiscible components in their free states, i.e., PSt and PDMS. Phase separation is clearly proved by DSC traces with two distinct glass transition temperatures in the regions of the PSt and PDMS homopolymers. The SAXS curves of all investigated conetworks have a maximum, indicating the nanophase-separated domain structure with *d*-spacing in the range of 8–17 nm. This decreases with increasing PDMS macrocrosslinker content, i.e., with increasing crosslinking density. The AFM phase images of cryo-sectioned surfaces corroborate the SAXS and DSC results by showing a disordered bicontinuous (cocontinuous) nanophasic morphology for all PSt-*l*-PDMS and PSt-*l*-PDMS/DVB conetworks, including even the hypercrosslinked conetworks with 1:5 (*m*/*m*) DVB/St ratio. The good resolution of the AFM images afforded to estimate the average domain distances, which fall in the range of 9–16 nm, i.e., in good agreement with the *d*-spacing values obtained by the SAXS measurements. These results of our systematic investigations clearly prove that the macroscopic phase separation is prevented by the covalent crosslinking between the immiscible PSt and PDMS chains, resulting in the formation of disordered bicontinuous nanophases in the PSt-*l*-PDMS and PSt-*l*-PDMS/DVB conetworks over the entire investigated compositional range with 30–80 wt% PDMS content. It can also be concluded that the high DVB content (1:5 DVB/St ratio) does not prevent either the incorporation of the MA-PDMS-MA macrocrosslinker or the nanophase separation between the styrenic and PDMS components in the hypercrosslinked conetworks.

The swelling in selective solvents, that is, 1-nitropropane (1-PrNO_2_) for PSt and hexane for PDMS, led to uniform swelling in both solvents, and this resulted in the corresponding organogels. This swelling behavior is in line with the bicontinuous nanophasic morphology. In other words, this finding provides additional evidence for the bicontinuous nanophase-separated structure of the PSt-*l*-PDMS and PSt-*l*-PDMS/DVB conetworks. The equilibrium swelling degrees (*Q*_e_) correlate well with the composition, i.e., *Q*_e_ increases with increasing PSt content in 1-PrNO_2_, and with increasing PDMS content in hexane for all conetworks. The addition of DVB decreases *Q*_e_, due to the increased crosslinking density. The diffusion exponents, determined from the swelling degree versus time curves by the Korsmeyer–Peppas relationship [113,114,115,116], fall below 0.45 indicating hindered Fickian diffusion swelling mechanism of the PSt-*l*-PDMS and PSt-*l*-PDMS/DVB conetworks in both 1-PrNO_2_ and hexane.

In sum, it can be concluded that radical copolymerization of styrene and methacrylate–telechelic PDMS, both in the absence and in the presence of DVB, results in conetworks with a bicontinuous disordered nanophasic morphology with domain sizes in the range of ~3–30 nm in a broad composition window with 30–80% (*m*/*m*) PDMS content. These conetworks are selectively swellable in appropriate solvents, resulting in special gels (organogels, hydrophobic gels, and oleogels) in which only one of the nanophases is swollen. This property can surpass organogels of homopolymer networks (see e.g., Refs. [117,118,119,120,121,122,123,124] and references therein) by the opportunity to have a much broader choice of organic solvents as swelling agents. Both the cocontinuous nanophasic morphology and the selective swelling behavior of the PSt-*l*-PDMS and PSt-*l*-PDMS/DVB conetworks can be utilized in various applications, ranging from optics to environmental protection, selective sorbents, catalyst supports, nanohybrids, specialty membranes, etc.

## 4. Materials and Methods

### 4.1. Materials

Styrene (St, 99%, Sigma-Aldrich, Steinheim, Germany) and divinylbenzene (DVB, 80% technical grade, consisting of 80% m/p-divinylbenzene and 20% ethylstyrene determined by ^1^H NMR, from Honeywell Fluka, Steinheim, Germany) were freshly distilled from calcium hydride under reduced pressure prior to use. Methacryloxypropyl-telechelic poly(dimethylsiloxane) (MA-PDMS-MA, Gelest Inc., Morrisville, PA, USA) was purified by precipitation of its hexane solution (20 *m*/*m*%) into methanol, then decanted and extracted with distilled water in a separatory funnel. The hexane fraction was dried on anhydrous magnesium sulfate, and after the filtration of the desiccant, hexane was evaporated by a rotary evaporator followed by drying the MA-PDMS-MA by vacuum at room temperature. This process led to the removal of the inhibitor from the commercial product as shown by comparing the ^1^H NMR spectra of the unpurified and purified polymer in Figures S5 and S6, respectively. The number average molecular weight (*M*_n_) of the purified MA-PDMS-MA was determined by ^1^H NMR (*M*_n_ = 4700 g/mol). α,α′-Azobisisobutyronitrile (AIBN, Sigma Aldrich, Steinheim, Germany) was recrystallized from methanol twice and dried under vacuum at room temperature prior to use. Tetrahydrofuran (THF, VWR International Ltd., Debrecen, Hungary) was refluxed over and distilled from sodium–benzophenone before use. Other chemicals, 1-nitropropane (1-PrNO_2_, Sigma Aldrich, Steinheim, Germany), n-hexane (Molar Chemicals, Halásztelek, Hungary), and methanol (MeOH, VWR International Ltd., Debrecen, Hungary) were used as received without further purification.

### 4.2. Synthesis of PSt-l-PDMS and PSt-l-PDMS/DVB Conetworks

The polystyrene-*l*-poly(dimethylsiloxane) (PSt-*l*-PDMS) conetworks were synthesized via free radical copolymerization of MA-PDMS-MA (*M*_n_ = 4700 g/mol) macromolecular crosslinker and styrene (St). The polystyrene-*l*-poly(dimethylsiloxane)/divinylbenzene (PSt-*l*-PDMS/DVB) type conetworks with additional DVB crosslinkers were prepared under the same condition. In both types of these conetworks, the PDMS content was varied between 30 and 70 *m*/*m*% in the feed. In the case of the PSt-*l*-PDMS/DVB conetworks, the St/DVB weight ratios were constant, 36:1 or 5:1. The feed compositions for the conetwork syntheses are presented in Tables S1–S3 in the Supporting Information. During the conetwork forming reactions, the ratio of the total monomer concentration ([M]) and the square root of the AIBN initiator concentration ([I]^1/2^) that is, [M]/[I]^1/2^, was 23.2 in all cases. The concentration of the reaction mixture was 0.4 g/mL. First, the appropriate amounts of starting materials and solvent (THF) were mixed and homogenized in a glovebox under nitrogen atmosphere. Then, the reaction mixtures were poured into disk-shaped Teflon molds. The molds were sealed, removed from the glovebox, and heated in an oven at 60 °C for 72 h. After the reaction, the molds were removed from the oven, allowed to cool to room temperature, and then opened to allow the THF to evaporate slowly.

For sample identification, each conetwork sample is denoted with capital letters representing the components, that is, styrene (“S”) and divinylbenzene (“D”). The number, following the letters, shows the St/DVB weight ratio. The middle number, separated by a dash (“-”), indicates the number average molecular weight of the MA-PDMS-MA in kg/mol. The last number specifies the PDMS content, expressed as *m*/*m*%.

The synthesized PSt-*l*-PDMS and PSt-*l*-PDMS/DVB conetworks were gently dried under vacuum at 130 °C for one day. Subsequently, the samples were purified by solvent extraction using distilled THF, hexane and 1-nitropropane (1-PrNO_2_) to remove the unreacted components and oligomers. During the extraction, the solvent was changed daily for three days. In the case of hexane and 1-PrNO_2_, the extraction was carried out separately with each solvent. The extraction residues of the samples were collected and analyzed by ^1^H NMR. Finally, the conetworks were dried by gradual heating in a vacuum oven until a constant weight was achieved. Before structural analyses, all the samples were annealed under vacuum at 130 °C for 24 h and then cooled slowly to room temperature in order to avoid thermal history effects.

### 4.3. Characterization

#### 4.3.1. ^1^H Nuclear Magnetic Resonance (^1^H NMR) Spectroscopy

Solution state ^1^H NMR spectra were recorded on a Varian iNOVA 500 spectrometer (Varian Inc., Palo Alto, CA, USA) operating at 500 MHz ^1^H frequency in deuterated chloroform at room temperature.

#### 4.3.2. Elemental Analysis

The elemental analysis (EA) was made on a Heraeus CHN-O-RAPID instrument (Heraeus, Hanau, Germany). The composition of the polymer conetworks was determined from the carbon content of the samples.

#### 4.3.3. Differential Scanning Calorimetry (DSC)

The DSC measurements were carried out by a Mettler Toledo DSC821e equipment (Mettler Toledo, MGreifensee, Switzerland) in the temperature range of zero to 200 °C with 10 °C/min heating/cooling rate under 80 mL/min nitrogen flow. The measurements in the temperature range of −150 to 0 °C were performed by a Perkin Elmer Diamond DSC (Perkin Elmer, Norwalk, CT, USA) instrument equipped with a liquid nitrogen cooling system with 10 °C/min heating/cooling rate with 20 mL/min helium flow. The glass transition temperature (*T*_g_) was determined as the inflection point of the DSC curves on the recorded second heating curves.

#### 4.3.4. Small Angle X-Ray Scattering (SAXS)

SAXS measurements were performed on the CREDO instrument [125] equipped with a GeniX3D Cu ULD beam delivery system with integrated FOX 3D parabolic multilayer optics (Xenocs SA, Sassenage, France) and a 30 W microfocus Cu anode X-ray tube (Xenocs SA, Sassenage, France). The produced X-ray beam (*λ* = 0.154 nm) was cut to the required cross-section and divergence using a 3-pinhole collimation scheme [126]. After interacting with the samples mounted on a ladder sample holder inside the evacuated sample chamber, scattered X-rays were detected with a Pilatus 300K CMOS hybrid pixel detector (Dectris Ltd., Baden, Switzerland). From each sample, several scattering patterns were recorded to assess the stability of the sample and the instrument. The sample-to-detector distances were nearly the same for each sample, covering the range of 0.05 < *q* < 2 nm^−1^, where the *q* is the magnitude of the scattering vector, defined as *q* = 4π sin θ/*λ*, where 2θ is the scattering angle and *λ* = 0.154 nm is the X-ray wavelength of the Cu Kα radiation used. The individual scattering patterns were subjected to the standard correction procedure of the instrument as soon as they were ready. Geometric effects, such as detector flatness, X-ray absorption in the sample, etc., were accounted for. The angular range was calibrated (through determining the sample-to-detector distance) using a mixture of silver behenate and a batch of SBA15 mesoporous silica which was previously calibrated in the same instrument on first principles basis. Although the intensity is routinely calibrated into absolute units of differential scattering cross-section using a piece of glassy carbon, due to the uncertainty of determining the sample thickness, in this case, we were only able to express the intensity on a relative scale of arbitrary units.

#### 4.3.5. Atomic Force Microscopy (AFM)

Phase imaging AFM measurements, which are widely used for morphology investigations of bi- and multicomponent polymer systems (blends, copolymers, networks; see e.g., Refs. [9,10,11,12,17,18,19,20,21,30,31,32,33,34,35,57,58,59,77,80,81,82,83,127,128,129,130,131] and references therein), were carried out with a MultiMode 8 Nanoscope instrument (BRUKER, Billerica, MA, USA) having a Nanoscope V controller in tapping mode and phase imaging at ambient conditions. New super-sharp silicon cantilever (SSS-NCLR, NANOSENSORS, Neuchatel, Switzerland), which assures a curvature of 2–5 nm radius, was used for the investigation. The SEM image of the cantilever tip used in this study is presented in Figure S7. The applied cantilevers can reproducibly scan up to 30 times 1 × 1 µm^2^ images. Prior to the experiments the polymer conetworks were cut to slices of 150 nm thickness using a diamond knife (Diatome, Nidau, Switzerland) at −120 °C in a Cryo-Ultramicrotom instrument (LEICA, Wien, Austria). After cryo-cutting, the samples were warmed to 35 °C in the cryo-chamber to avoid water condensation on the freshly cut surfaces. After removing from the warmed-up cryo-chamber, the freshly cut surface was examined by AFM within 30 min. The evaluation of the AFM phase images for the estimation of the phase distances was carried out by using the distance tool of the free and open Gwyddion software (Version 2.53) for 2D data analysis of microscopy images. For each image, the average domain distances were obtained by averaging minimum sixty distance values between the two different phases from randomly selected spots in the AFM images.

#### 4.3.6. Swelling Measurements

Swelling experiments were carried out by immersing the conetwork samples in large excess of 1-PrNO_2_ and n-hexane at ambient temperature. Then, the weight of the samples was measured at predetermined time intervals until the samples reached the equilibrium swelling degree (*Q*_e_). The swelling degrees (*Q*) were calculated by the following formula:

(3) $$Q={(m}_{s}-m_{d})/m_{d}$$

where *m*_d_ and *m*_s_ are the weight of the dry and the swollen samples at a given swelling time, respectively.

## Figures and Tables

**Table 1:** The PDMS contents of the feed, the gel fraction and the composition of the PSt-*l*-PDMS and the PSt-*l*-PDMS/DVB conetworks, determined by elemental analysis (*m*_PDMS,EA_) and by the composition of the extractables residues (*m*_PDMS,extr_) obtained by the ^1^H NMR spectra of the extraction residues (see Section 4.2 for sample identification).

Sample ID	*m*_PDMS,feed_ (%)	Gel Fraction (%)	*m*_PDMS,EA_ by Elemental Analysis (%)	*m*_PDMS,extr_ by Composition of Extractables (%)
S-4.7-48	30	55.9	n. a.	48.4
S-4.7-59	40	60.4	61.3	59.3
S-4.7-67	50	60.2	67.1	67.4
S-4.7-76	60	63.0	77.2	76.4
S-4.7-84	70	53.2	84.1	n. a.
SD36-4.7-33	30	88.1	n. a.	33.4
SD36-4.7-44	40	86.3	n. a.	44.3
SD36-4.7-55	50	86.7	n. a.	55.4
SD36-4.7-65	60	87.1	n. a.	65.2
SD36-4.7-75	70	86.8	n. a.	75.4
SD5-4.7-32	30	92.1	33.8	32.4
SD5-4.7-43	40	88.9	42.3	43.4
SD5-4.7-54	50	87.8	52.6	53.9
SD5-4.7-63	60	85.9	61.3	62.7
SD5-4.7-75	70	84.1	73.6	75.0

n. a. = not available.

**Table 2:** The *d*-spacing by SAXS and the average domain distances (*d*) by AFM in the PSt-*l*-PDMS and PSt-*l*-PDSM/DVB conetworks.

Sample ID	*d*-Spacing (nm) by SAXS	*d* (nm) by AFM
S-4.7-48	16.7	16
S-4.7-59	14.1	14
S-4.7-67	12.8	13
S-4.7-76	11.7	11
S-4.7-84	11.7	12
SD36-4.7-33	11.9	15
SD36-4.7-44	11.0	15
SD36-4.7-55	10.1	14
SD36-4.7-65	9.5	13
SD36-4.7-75	8.3	12
SD5-4.7-32	11.0	11
SD5-4.7-43	10.4	10
SD5-4.7-54	9.5	9
SD5-4.7-63	8.5	9
SD5-4.7-75	7.7	10

## Data Availability

The original contributions presented in this study are included in the article/Supplementary Materials. Further inquiries can be directed to the corresponding author.

## Supplementary Materials

The following supporting information can be downloaded at: https://www.mdpi.com/article/10.3390/gels11050318/s1, List of abbreviations. Table S1. The feed amounts for the preparation of the PSt-*l*-PDMS conetworks (solvent: THF, total volume: 3.75 mL). Table S2. The feed amounts for the preparation of the PSt-*l*-PDMS/DVB conetworks (the St/DVB weight ratio is 36:1, solvent: THF, total volume: 5 mL). Table S3. The feed amounts for the preparation of the PSt-*l*-PDMS/DVB conetworks (the St/DVB weight ratio is 5:1, solvent: THF, total volume: 5 mL). Figure S1. The swelling degrees of the PSt-*l*-PDMS and the PSt-*l*-PDMS/DVB conetworks in 1-nitropropane as a function of time. Figure S2. The swelling degrees of the PSt-*l*-PDMS and the PSt-*l*-PDMS/DVB conetworks in n-hexane as a function of time. Figure S3. The equilibrium swelling degrees of PSt-*l*-PDMS and PSt-*l*-PDMS/DVB conetworks in 1-nitropropane (left) and in n-hexane (right). Figure S4. The double logarithmic plot of the swelling degrees and time in the initial stage of swelling according to Korsmeyer–Peppas equation for the PSt-*l*-PDMS and PSt-*l*-PDMS/DVB conetworks. Figure S5. The ^1^H NMR spectrum of MA-PDMS-MA in CDCl_3_ before the purification. The circled values belong to the BHT inhibitor (The framed value belongs to water). Figure S6. The ^1^H NMR spectrum of the purified MA-PDMS-MA in CDCl_3_. The BHT signals cannot be detected (The framed value belongs to water). Figure S7. The SEM image of a new SSS cantilever. The curvature radius is estimated to be 5 nm.
