# Supplementary material for: Bicontinuous Nanophasic Conetworks of Polystyrene with Poly(dimethylsiloxane) and Divinylbenzene: From Macrocrosslinked to Hypercrosslinked Double-Hydrophobic Conetworks and Their Organogels with Solvent-Selective Swelling
Source: Gels. 2025 Apr 24;11(5):318. doi: 10.3390/gels11050318 (PMC12110945; doi:10.3390/gels11050318)
Supplement: Supplementary file 1 [file gels-11-00318-s001.zip › gels-3593097-supplementary.pdf]

SUPPLEMENTARY MATERIAL

Bicontinuous nanophasic polystyrene-*l*-poly(dimethylsiloxane) and polystyrene-*l*-poly(dimethylsiloxane)/divinylbenzene double-hydrophobic conetworks: from macrocrosslinked to hypercrosslinked conetworks and their organogels with solvent selective swelling behavior

Anna Petróczy[1,2], István Szanka[1], András Wacha[3], Zoltán Varga[3], Yi Thomann[4,5], Ralf Thomann[4,5], Rolf Mülhaupt[4,5,6], Laura Bereczki[7], Nóra Hegyesi[1,8], Béla Iván[1*]

[1] Polymer Chemistry and Physics Research Group, Institute of Materials and Environment Chemistry, HUN-REN Research Centre for Natural Sciences, Magyar tudósok körútja 2, H-1117 Budapest, Hungary

[2] George Hevesy PhD School of Chemistry, Institute of Chemistry, Faculty of Science, Eötvös Loránd University, Pázmány Péter sétány 2, H-1117 Budapest, Hungary

[2] Biological Nanochemistry Research Group, Institute of Materials and Environmental Chemistry, HUN-REN Research Centre for Natural Sciences, Magyar tudósok körútja 2, H-1117 Budapest, Hungary

[4] Freiburg Materials Research Center, University of Freiburg, Stefan-Meier-Str. 21, D-79104 Freiburg, Germany

[5] Freiburg Center for Interactive Materials and Bioinspired Technologies (FIT), University of Freiburg, Georges-Köhler-Allee 105, D-79110 Freiburg, Germany

[6] Institute for Macromolecular Chemistry, University of Freiburg, Stefan-Meier-Str. 31, D-79104 Freiburg, Germany

[7] Chemical Crystallography Research Laboratory. Centre of Structural Science, HUN-REN Research Centre for Natural Sciences, Magyar tudósok körútja 2, H-1117 Budapest, Hungary

[8] Department of Physical Chemistry and Materials Science, Budapest University of Technology and Economics, Műegyetem rkp. 3, H-1111 Budapest, Hungary

***** Correspondence: ivan.bela@ttk.hu (B.I.)

List of abbreviations

AFM	Atomic Force Microscopy
AIBN	α,α'-Azobisisobutyronitrile
BHT	Butylated hydroxytoluene
d	*d*-spacing (average domain distance)
DSC	Differential Scanning Calorimetry
DVB	Divinylbenzene
HEA	2-Hydroxyethyl acrylate
-l-	*-linked by-*
MA-PDMS-MA	Methacryloxypropyl-telechelic poly(dimethylsiloxane)
NMR	Nuclear Magnetic Resonance
PDMS	Poly(dimethylsiloxane)
PEG	Poly(ethylene glycol)
PHEA	Poly(2-hydroxyethyl acrylate)
1-$PrNO_2$	1-Nitropropane
$^{i}PrOH$	Isopropyl alcohol
PSt	Polystyrene
Q_e	Equilibrium swelling degree
SAXS	Small Angle X-ray Scattering
St	Styrene
T_g	Glass transition temperature
THF	Tetrahydrofuran

Table S1. The feed amounts for the preparation of the PSt-*l*-PDMS conetworks (solvent: THF, total volume: 3.75 ml).

Sample ID	PDMS (m/m%)	PDMS_4.7k *m* (g)	AIBN *m* (mg)	St *V* (ml)
S-4.7-48	30	0.4322	6.41	1.159
S-4.7-59	40	0.6235	4.81	0.993
S-4.7-67	50	0.7501	3.43	0.828
S-4.7-76	60	0.8916	2.29	0.662
S-4.7-84	70	1.0421	1.38	0.497

Table S2. The feed amounts for the preparation of the PSt-*l*-PDMS/DVB conetworks (the St/DVB weight ratio is 36:1, solvent: THF, total volume: 5 ml).

Sample ID	PDMS (m/m%)	PDMS_4.7k *m* (g)	AIBN *m* (mg)	St *V* (ml)	DVB *V* (μl)
SD36-4.7-33	30	0.6063	11.66	1.503	41.4
SD36-4.7-44	40	0.7844	8.74	1.289	35.5
SD36-4.7-55	50	0.9872	6.25	1.074	29.6
SD36-4.7-65	60	1.1837	4.17	0.859	23.7
SD36-4.7-75	70	1.3888	2.51	0.644	17.7

Table S3. The feed amounts for the preparation of the PSt-*l*-PDMS/DVB conetworks (the St/DVB weight ratio is 5:1, solvent: THF, total volume: 5 ml).

Sample ID	PDMS (m/m%)	PDMS_4.7k *m* (g)	AIBN *m* (mg)	St *V* (ml)	DVB *V* (ml)
SD5-4.7-32	30	0.5965	13.08	1.288	0.255
SD5-4.7-43	40	0.7819	9.80	1.104	0.219
SD5-4.7-54	50	0.9753	6.99	0.919	0.183
SD5-4.7-63	60	1.1988	4.65	0.736	0.146
SD5-4.7-75	70	1.3911	2.79	0.552	0.109

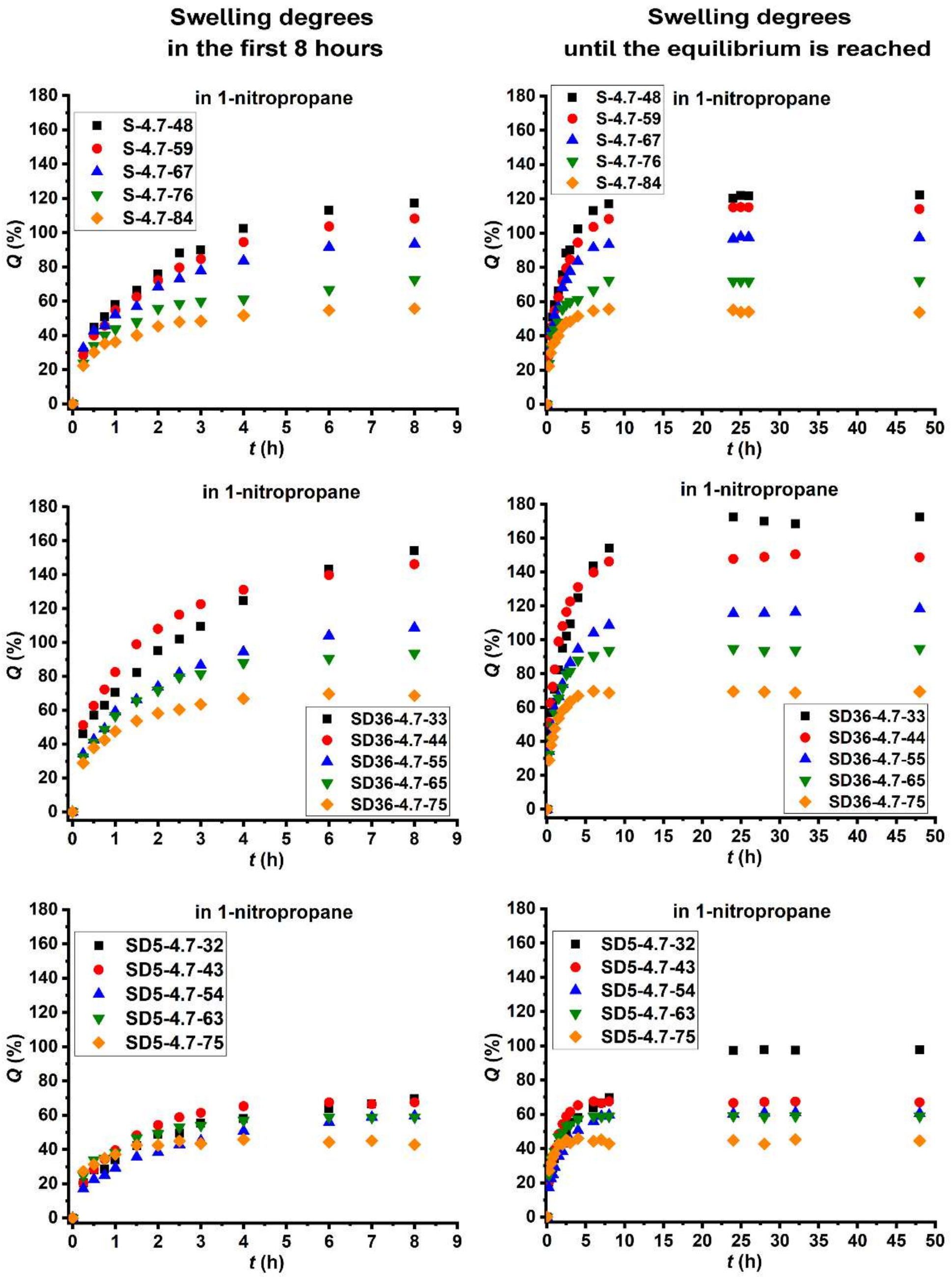

Figure S1. The swelling degrees of the PSt-*l*-PDMS and the PSt-*l*-PDMS/DVB conetworks in 1-nitropropane as a function of time.

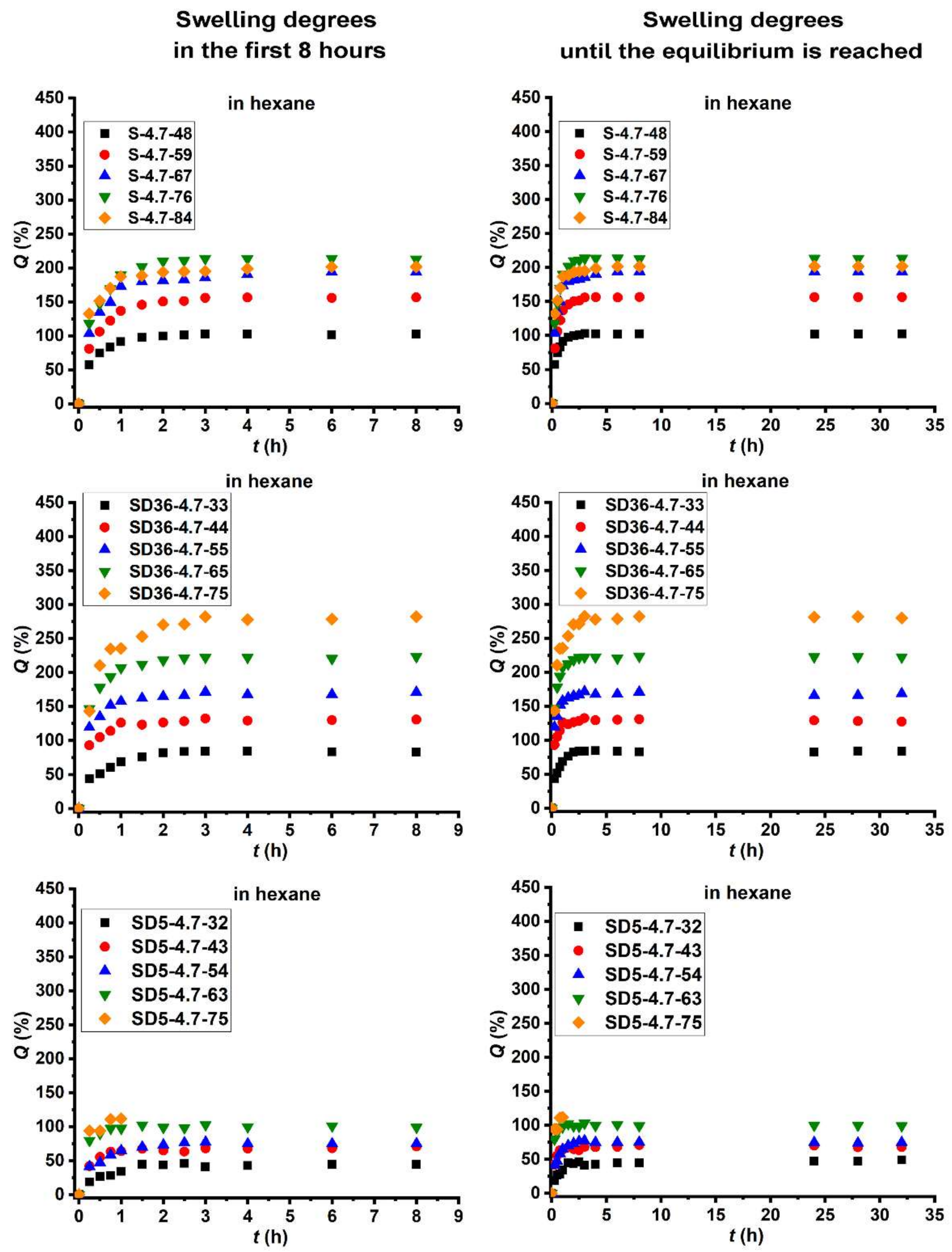

Figure S2. The swelling degrees of the PSt-*l*-PDMS and the PSt-*l*-PDMS/DVB conetworks in n-hexane as a function of time.

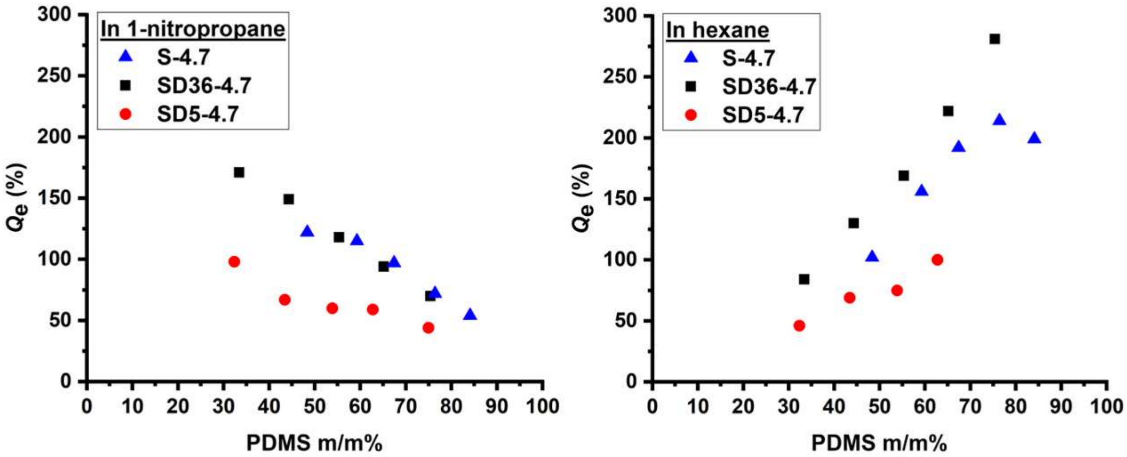

Figure S3. The equilibrium swelling degrees of PSt-*l*-PDMS and PSt-*l*-PDMS/DVB conetworks in 1-nitropropane (left) and in n-hexane (right) as a function of the PDMS content.

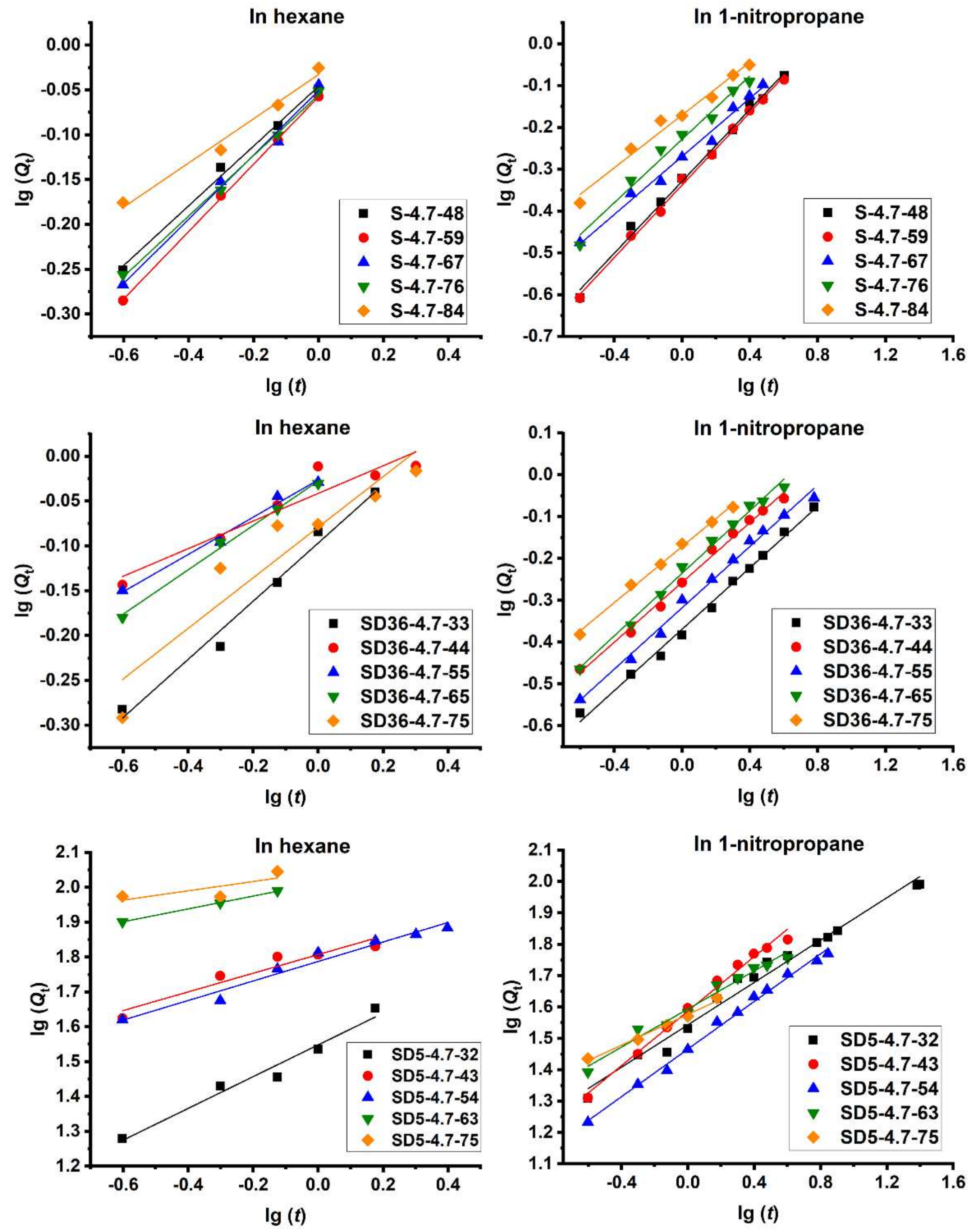

Figure S4. The double logarithmic plot of the swelling degrees and time in the initial stage of swelling according to the Korsmeyer-Peppas equation for the PSt-*l*-PDMS and PSt-*l*-PDMS/DVB conetworks.

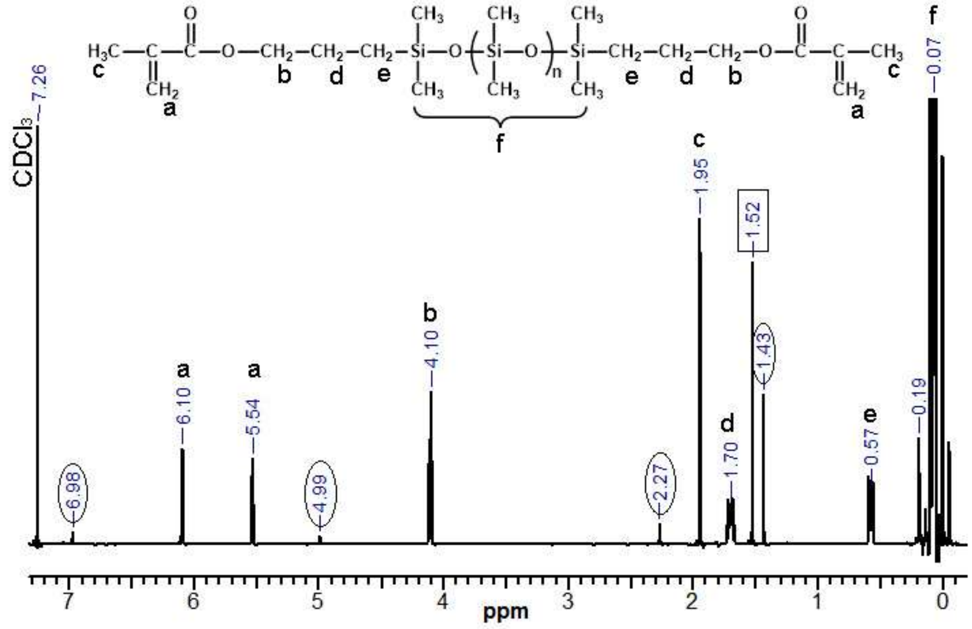

Figure S5. The ^1H NMR spectrum of MA-PDMS-MA in $CDCl_3$ before the purification. The circled values belong to the BHT inhibitor. (The framed value belongs to water.)

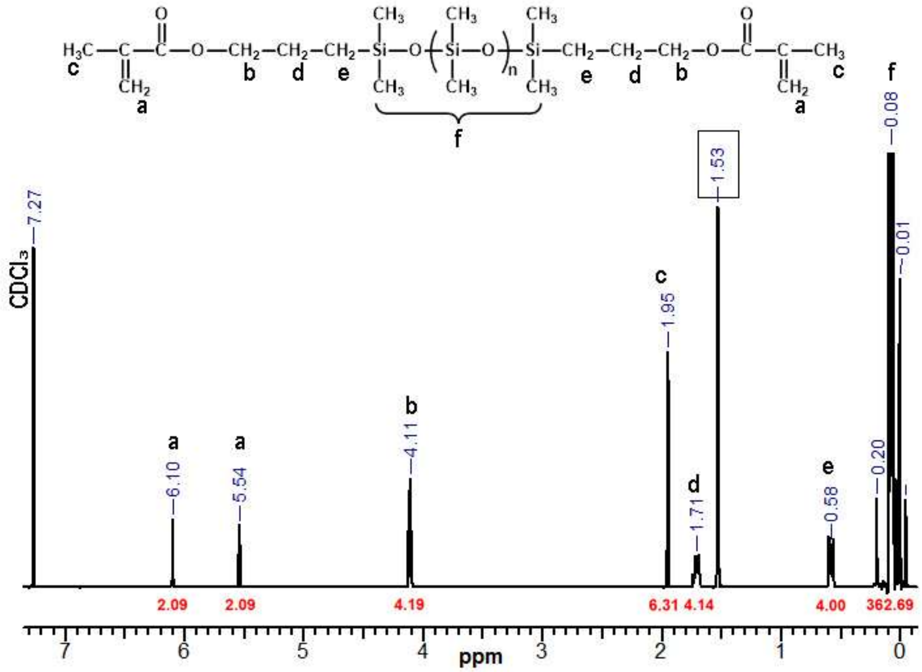

Figure S6. The ^1H NMR spectrum of the purified MA-PDMS-MA in $CDCl_3$. The BHT signals cannot be detected. (The framed value belongs to water.)

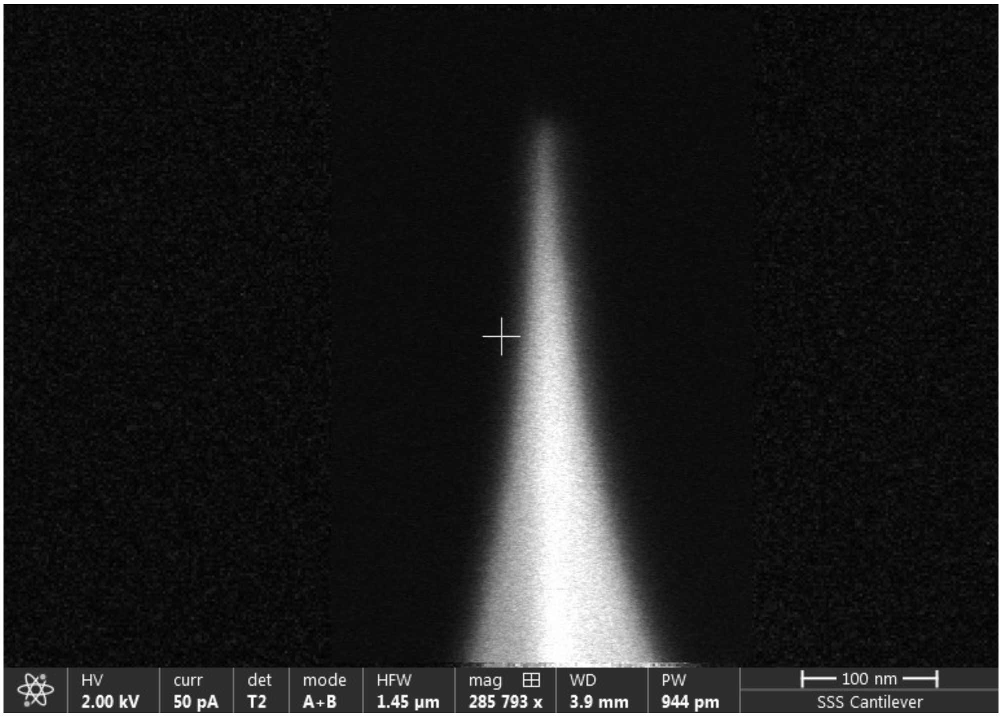

Figure S7. The SEM image of a new SSS cantilever. The curvature radius is estimated to be 5 nm.
